# Increased Intestinal Permeability and Articular Involvement in Systemic Lupus Erythematosus Patients—A Mutually Exclusive Relationship?

**DOI:** 10.3390/cimb47110922

**Published:** 2025-11-05

**Authors:** Cristian-Mihai Ilie, Cătălina-Anamaria Boromiz, Irina Anna-Maria Stoian, Laura Elena Gaman, Laura Groșeanu, Andra Rodica Bălănescu, Marilena Gîlcă

**Affiliations:** 1 Department of Functional Sciences I/Biochemistry, Carol Davila University of Medicine and Pharmacy, 050474 Bucharest, Romania; cristian-mihai.ilie@rez.umfcd.ro (C.-M.I.); irina.stoian@umfcd.ro (I.A.-M.S.); laura.gaman@umfcd.ro (L.E.G.); 2 Department of Rheumatology and Internal Medicine, Sfânta Maria Clinical Hospital, 011172 Bucharest, Romania; catalinaboromiz@gmail.com (C.-A.B.); laura_groseanu@yahoo.com (L.G.); balanescu.andra@gmail.com (A.R.B.); 3 Department of Rheumatology, Carol Davila University of Medicine and Pharmacy, 011172 Bucharest, Romania

**Keywords:** zonulin, leaky gut, intestinal permeability, systemic lupus erythematosus, articular

## Abstract

Systemic lupus erythematosus (SLE) is a multisystemic autoimmune disorder characterized by complex interactions between the innate and adaptive immune systems, being potentially associated with an enhanced intestinal permeability. Zonulin represents a key protein in the modulation of intestinal permeability, being a gut leakage marker. The purpose of the present work was to evaluate the intestinal permeability, through serum zonulin levels, and to explore the relationships between zonulin, disease activity, and organ involvement in Caucasian SLE patients. The study had a cross-sectional design and included two groups of subjects: the SLE group (*n* = 41) and the control group (*n* = 29). Plasma zonulin level was measured using indirect ELISA. Despite the fact that Caucasian SLE patients exhibited higher plasma zonulin levels compared to the control group (7.566 ± 1.368 ng/mL vs. 2.306 ± 0.286 ng/mL, *p* < 0.01, Mann–Whitney-*U*-test), plasma zonulin levels did not correlate with disease activity measured by the Systemic Lupus Erythematosus Disease Activity Index (SLEDAI). SLE patients with clinical articular involvement had paradoxically lower plasma zonulin levels than those without this manifestation. The results support the hypothesis of a mutually exclusive inflammatory “signature” between intestinal mucosa and synovium.

## 1. Introduction

Systemic lupus erythematosus (SLE) is a persistent autoimmune disease characterized by a broad spectrum of clinical manifestations and a pattern of episodic flare-ups and remissions [1]. Complex interactions between the innate and adaptive immune systems, genetic and environmental factors contribute to the etiology and pathogenesis of SLE [1,2].

A population-based surveillance study from European countries showed that the median age at SLE diagnosis was 30 years, with a median diagnosis delay of 2 years [3]. The organ systems most frequently affected were the joints (81.8%), skin (59.4%), and kidneys (30%) [3]. SLE exhibits a pronounced gender disparity, affecting women at a rate nine times higher than that of men [4].

Long-term exposure of the intestinal surface to antigens (commensal microbes that reside in the intestinal lumen, foodborne pathogens, and food antigens) is responsible for the development of barrier functions by intestinal epithelial cells, which, thus, restrict the entry of external antigens from the intestinal lumen to the submucosa and prevent the escape of endogenous substances, maintaining the biological homeostasis [5].

An imbalanced diet, excessive alcohol consumption, stress, exposome (exposure to various environmental pollutants including heavy metals, pesticides, microplastics, detergents, surfactants, shampoos, body cleaners, etc.), as well as the use of antibiotics and other medications, can alter the composition of the intestinal microbiota and compromise the integrity of the intestinal barrier [6,7,8]. This leads to an increased intestinal permeability (“leaky gut”), which allows the translocation of harmful agents across the epithelial junctions into the bloodstream. Consequently, a chronic inflammatory state is induced, and this negatively impacts multiple organs and systems [9].

In an SLE mouse model study, induced gut leakage facilitated the translocation of microbial components into systemic circulation, promoting systemic inflammation (evidenced by elevated serum IL-6 levels), increased anti-dsDNA antibody production, and immune-complex depositions, thereby exacerbating SLE progression and disease activity [10]. Therefore, it would be a reasonable presumption that hyperactivation of the immune system secondary to altered intestinal permeability could contribute to various organ involvements, including articular manifestations, in SLE.

Literature data indicate that leaky gut syndrome is present in the majority, if not all, patients with SLE [11]. The detection of bacteria and bacterial components in the blood of SLE patients supports the association with leaky gut syndrome. Studies have shown that SLE patients display significantly elevated circulating levels of lipopolysaccharides (LPS), or endotoxins, in comparison with healthy controls. This increase in blood microbial components has been implicated in the induction of aberrant gene expression patterns, thereby contributing to the complex immune dysregulation (e.g., autoantibody development) observed in SLE pathogenesis [12,13].

Zonulin, a molecule identified in 2000, produced primarily in the small intestine, plays a crucial role in regulating the integrity of the intestinal barrier through the modulation of tight junctions in intestinal epithelial cells [14,15]. In 2009, it was identified as pre-haptoglobin 2 [16]. Studies have shown that increased intestinal permeability is associated with elevated zonulin expression [17]. Small intestinal exposure to bacteria and gluten is an example of a powerful trigger of zonulin release from the gut [14].

The aim of the study was to investigate the relationship between intestinal permeability, assessed by plasma zonulin levels, and disease activity, assessed by the Systemic Lupus Erythematosus Disease Activity Index—SLEDAI. Furthermore, the study aimed to examine the relationship between intestinal permeability and organ damage in SLE patients, its potential correlations with various routine hematological, immunological, and biochemical parameters (e.g., erythrocyte sedimentation rate—ESR, C-reactive protein—CRP, white blood cells—WBC, lymphocytes—LYM, thrombocytes—PLT, hemoglobin—Hb, creatinine, anti-double stranded DNA antibodies—anti-dsDNA ab, complement component 3—C3 plasma level, complement component 4—C4 plasma level, and serum uric acid), as well as the impact of medication on intestinal permeability in patients with SLE.

## 2. Materials and Methods

This cross-sectional study was conducted between January 2024 and May 2024 in “Sfânta Maria” Clinical Hospital, Bucharest, Romania.

A total of 70 participants were recruited for the study, including 41 patients with SLE and 29 healthy subjects. Both the patients with SLE and healthy controls are Romanian citizens. The inclusion criteria for the SLE group were age over 18 years and patients classified according to the 2019 ACR/EULAR classification criteria [18]. Clinical articular involvement was defined as tenderness and swelling affecting at least two peripheral joints, consistent with nonerosive inflammatory arthritis. The control group comprised adults over 18 years old who were screened and confirmed to be free of autoimmune or autoinflammatory disorders. Exclusion criteria for both groups of patients encompassed a documented history of infectious or acute cardiovascular events within the last three months, any concomitant autoimmune, autoinflammatory, or inflammatory disorders, or active malignancies.

The main variable was defined as serum zonulin level, and all subjects underwent blood sampling to determine the level of serum zonulin, which was measured using the enzyme-linked immunosorbent assay (ELISA) method, in order to assess the level of intestinal permeability.

Demographic characteristics (age, gender, place of origin), smoking status, and presence of anxiety/depression (as diagnosed by a psychiatrist) were recorded at the enrollment visit for both patients with SLE and the control group (Table 1).

Clinical characteristics and laboratory parameters (different organ involvement, SLEDAI, presence of secondary Sjogren’s syndrome, disease duration since symptom onset, disease duration since diagnosis, ESR, CRP, WBC, LYM, PLT, Hb, creatinine, anti-dsDNA ab, C3 and C4 plasma levels, and serum uric acid) were collected from patients with SLE.

Each participant underwent a single visit. The visits were organized in accordance with routine follow-up appointments of the patients at the hospital. The patients visiting for their routine check-up were approached and requested consent to participate in the study during their scheduled visits. In addition to the standard clinical and biological evaluation, all subjects underwent blood sampling (4 mL) into EDTA K2-containing tubes after an overnight fast, with the specific purpose of measuring serum zonulin levels. After centrifugation for 15 min at 10000× *g*, the plasma was stored at −80 °C before the zonulin assay.

Plasma level of zonulin was measured using an enzyme-linked immunosorbent assay kit (Human Zonulin ELISA 96T Cusabio, Wuhan, China). The assays were carried out on duplicate samples, and the results were read on the Mindray Mr-96A microplate reader (Shenzhen Mindray Bio-Medical Electronics Co., Ltd., Shenzhen, China). The inter-assay coefficient of variation fulfilled the requirements given in the manufacturer’s instructions. The mean values were utilized for data analysis.

Ethical approval was obtained from the Ethics Committee of “Sfânta Maria” Clinical Hospital (no. 30010 from 12 May 2023) and all procedures were carried out in accordance with the Declaration of Helsinki. All patients provided written informed consent to participate in the study.

Statistical analyses were performed using the GraphPad Prism V10. Nonparametric methods were used for statistical analysis of the data, as plasma zonulin level was not normally distributed. Correlations were made by Spearman’s test. Comparisons between two independent groups were conducted using the Mann–Whitney *U*-test. For comparisons of three or more independent groups, the Kruskal–Wallis test was used. Levels of statistical significance were set at *p* < 0.05.

## 3. Results

Clinical characteristics and laboratory parameters (different organ involvement, SLEDAI, presence of secondary Sjogren’s syndrome, disease duration since symptom onset, disease duration since diagnosis, plasma zonulin level, ESR, CRP, WBC, LYM, PLT, Hb, creatinine, anti-dsDNA ab, C3 and C4 plasma levels, and serum uric acid) for patients with SLE are shown in Table 2 and Table 3.

Between the two groups, no statistically significant difference was observed regarding gender (*p* = 0.37), age (*p* = 0.076), place of origin (*p* = 0.065), smoking status (*p* = 0.683), or the incidence of anxiety or depression (*p* = 0.1) (Table 1).

### 3.1. Intestinal Barrier Integrity in SLE Patients vs. Control Group

**Figure 1:**
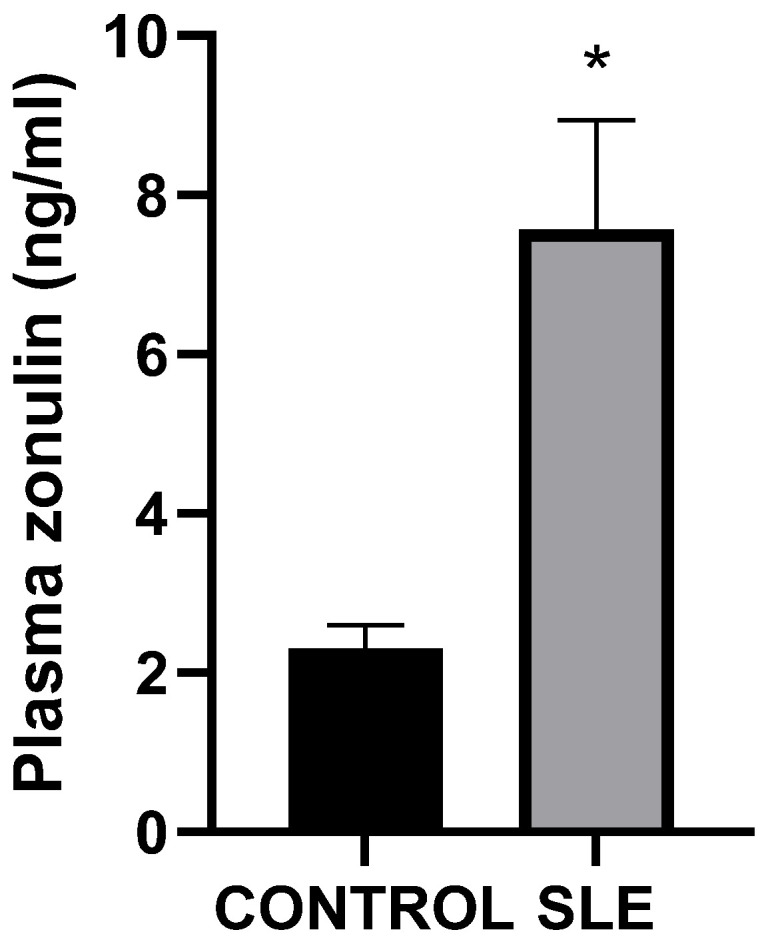
Plasma zonulin (ng/mL, mean ± SEM) SLE vs. Control (*n* = 41, 7.566 ± 1.368 vs. *n* = 29, 2.306 ± 0.286, *p* < 0.01, Mann–Whitney *U*-test), * *p* < 0.01.

The level of plasma zonulin in the SLE group was significantly higher than in the healthy control group (*n* = 41, 7.566 ± 1.368 vs. *n* = 29, 2.306 ± 0.286, *p* < 0.01; Figure 1), indicating that SLE patients have significantly increased intestinal permeability compared to the control group. Considering that the patients with SLE exhibited significantly higher values of plasma zonulin compared to the control group, further statistical analysis was conducted within this cohort. Thus, there were no statistically significant differences in plasma zonulin levels between patients from rural areas and those from urban settings (*n* = 16, 6.861 ± 2.553 vs. *n* = 25, 8.018 ± 1.578, *p* = 0.197), nor between smokers, former smokers, and non-smokers (*n* = 7, 5.437 ± 1.499 vs. *n* = 6, 11.75 ± 4.097 vs. *n* = 28, 7.202 ± 1.756, *p* = 0.411). Plasma zonulin levels were also not correlated with the age of patients with SLE (r = 0.298, *p* = 0.057).

### 3.2. Relationship Between Intestinal Permeability and Laboratory Parameters of SLE Patients

**Figure 2:**
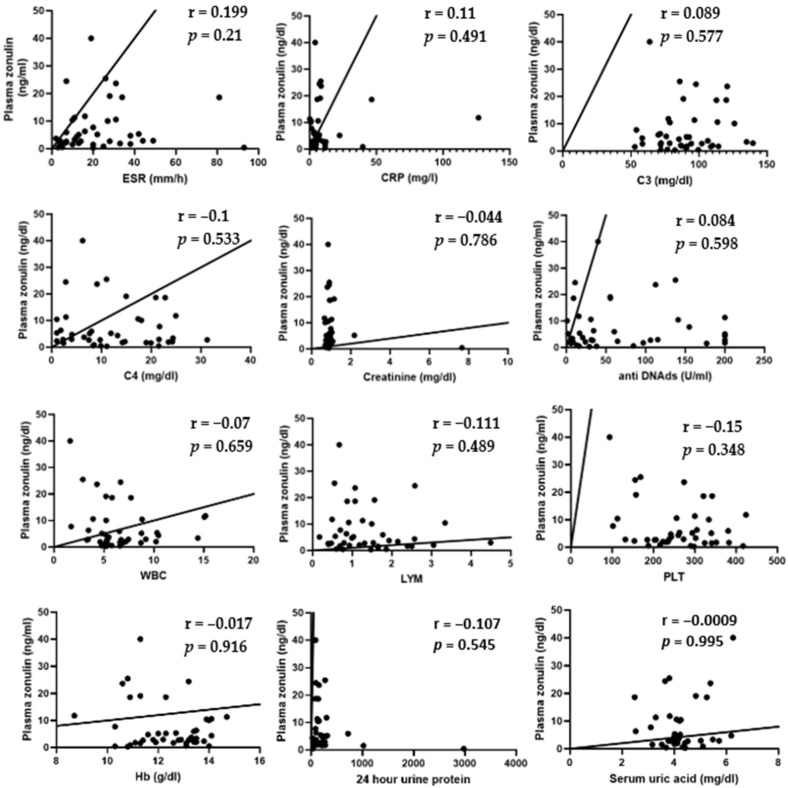
Correlation between plasma zonulin and ESR (r = 0.199; *p* = 0.21), CRP (r = 0.11; *p* = 0.491), C3 (r = 0.089; *p* = 0.577), C4 (r = −0.1; *p* = 0.533), creatinine (r = −0.044, *p* = 0.786), anti-dsDNA ab (r = 0.084; *p* = 0.598), WBC (r = −0.07; *p* = 0.659), LYM (r = −0.111; *p* = 0.489), PLT (r = −0.15; *p* = 0.348), Hb (r = −0.017, *p* = 0.916), 24 h urine protein (r = −0.107; *p* = 0.545), and uric acid level (r = −0.0009; *p* = 0.995)—Spearman’s test.

Spearman’s test showed no correlation between plasma zonulin and ESR (r = 0.199; *p* = 0.21), CRP (r = 0.11; *p* = 0.491), C3 (r = 0.089; *p* = 0.577), C4 (r = −0.1; *p* = 0.533), creatinine (r = −0.044, *p* = 0.786), anti-dsDNA ab (r = 0.084; *p* = 0.598), WBC (r = −0.07; *p* = 0.659), LYM (r = −0.111; *p* = 0.489), PLT (r = −0.15; *p* = 0.348), Hb (r = −0.017, *p* = 0.916), 24 h urine protein (r = −0.107; *p* = 0.545), and uric acid level (r = −0.0009; *p* = 0.995). These results suggest that intestinal permeability does not correlate with the level of inflammatory syndrome, serum complement levels, creatinine, uric acid, anti-dsDNA antibody levels, 24 h urine protein levels, or the blood count in patients with SLE (Figure 2).

### 3.3. Relationship Between Intestinal Permeability and Disease Activity in SLE Patients

**Figure 3:**
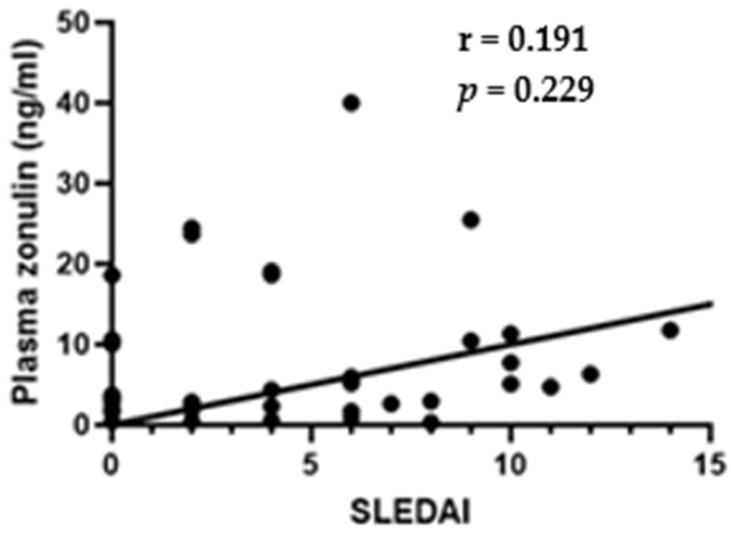
Correlation between plasma zonulin and SLEDAI (r = 0.191; *p* = 0.229)—Spearman’s test.

Spearman’s test showed no correlation between plasma zonulin and SLEDAI (r = 0.191; *p* = 0.229), indicating that intestinal permeability does not correlate with disease activity (Figure 3).

Furthermore, in the context of disease activity, SLE patients were divided into two groups based on SLEDAI score of <6 and ≥6; nevertheless, no statistically significant differences in plasma zonulin levels were detected between these groups (*n* = 22, 7.235 ± 1.727 vs. *n* = 19, 7.951 ± 2.221, *p* = 0.549, Mann–Whitney *U*-test). Additionally, SLE patients were stratified based on their anti-dsDNA antibody levels, with groups defined by values below and above twice the upper limit of the normal range; however, no statistically significant difference in plasma zonulin level was observed between these groups (*n* = 16, 5.915 ± 1.744 vs. *n* = 25, 8.623 ± 1.945, *p* = 0.307, Mann–Whitney *U*-test). In a similar manner, no statistically significant difference in plasma zonulin level was observed between SLE patients with hypocomplementemia and those with normal serum complement values (*n* = 22, 7.257 ± 2.054 vs. *n* = 19, 7.924 ± 1.807, *p* = 0.613, Mann–Whitney *U*-test).

Regarding disease duration, plasma zonulin levels had no correlation with either the time since the disease onset (r = −0.289, *p* = 0.063, Spearman’s test) or the time since diagnosis (r = −0.205, *p* = 0.191, Spearman’s test).

### 3.4. Relationship Between Intestinal Permeability and Clinical Findings in SLE Patients

**Figure 4:**
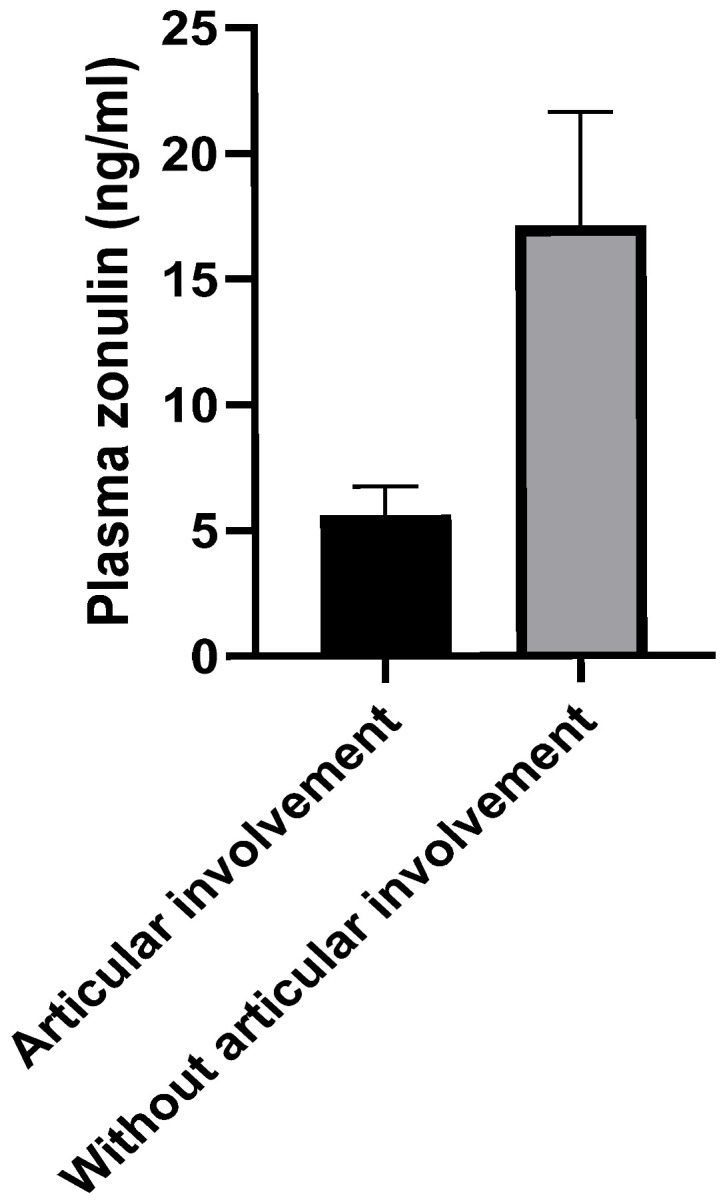
Plasma zonulin level (ng/mL, mean ± SEM) in SLE patients without articular involvement compared to those with articular involvement (*n* = 7, 17.1 ± 4.529 vs. *n* = 34, 5.604 ± 1.134, *p* < 0.01, Mann–Whitney *U*-test).

Regarding the clinical articular involvement in SLE, a statistically significantly higher level of plasma zonulin was observed in patients without articular impairment compared to those with joint manifestations (*n* = 7, 17.1 ± 4.529 vs. *n* = 34, 5.604 ± 1.134, *p* < 0.01; Figure 4).

According to Mann–Whitney-*U*-test, except the articular involvement, no statistically significant differences regarding organ impairment were observed, as follows: renal damage vs. without renal damage (*n* = 11, 6.595 ± 2.791 vs. *n* = 30, 7.923 ± 1.590, *p* = 0.237), neurological disability vs. without neurological disability (*n* = 8, 7.478 ± 2.16 vs. *n* = 33, 7.588 ± 1.631, *p* = 0.446), hematological ailment vs. without hematological ailment (*n* = 30, 8.855 ± 1.781 vs. *n* = 11, 4.051 ± 1.065, *p* = 0.183), history of serositis vs. without history of serositis (*n* = 10, 6.633 ± 2.321 vs. *n* = 31, 7.867 ± 1.664, *p* = 0.822) and cutaneous involvement vs. without cutaneous involvement (*n* = 26, 5.861 ± 1.204 vs. *n* = 15, 10.52 ± 3.025, *p* = 0.445).

No statistically significant difference was observed in the plasma zonulin levels between patients with SLE and secondary Sjogren’s syndrome and those without secondary Sjogren’s syndrome (*n* = 22, 7.945 ± 1.631 vs. *n* = 19, 7.127 ± 2.317, *p* = 0.343).

No statistically significant difference in plasma zonulin level has been identified between individuals affected by anxiety and/or depression and those without (*n* = 9, 8.506 ± 2.205 vs. *n* = 33, 7.170 ± 1.607, *p* = 0.21, Mann–Whitney *U*-test).

### 3.5. Relationship Between Intestinal Permeability and Medications Used in the Treatment of SLE

In relation to vitamin D supplementation, no statistically significant differences in plasma zonulin levels were observed between SLE patients receiving vitamin D and those not receiving it (*n* = 35, 6.627 ± 1.260 vs. *n* = 6, 13.05 ± 5.683, *p* = 0.183). Likewise, vitamin C supplementation did not yield statistically significant differences in plasma zonulin levels between patients who received vitamin C and those who did not (*n* = 3, 2.04 ± 0.745 vs. *n* = 39, 8.003 ± 1.453, *p* = 0.213).

No statistically significant difference in plasma zonulin level was found between patients who received glucocorticoids (GCs) and those who did not (*n* = 27, 7.588 ± 1.782 vs. *n* = 14, 7.525 ± 2.151, *p* = 0.734).

In the group of patients not undergoing glucocorticoid therapy (*n* = 14), although the patients treated with hydroxychloroquine (HCQ) combined with azathioprine (AZA) had the highest mean zonulin levels (*n* = 4, 13.66 ± 6.323), there was no statistically significant difference (*p* = 0.329) between the subgroup treated with HCQ (*n* = 5, 4.326 ± 1.561) and the subgroup treated with Belimumab (*n* = 2, 1.61 ± 1.12).

In the group of patients treated with GCs (*n* = 27), no statistically significant differences (*p* = 0.631) were observed in plasma zonulin levels. SLE patients with HCQ administration exhibited higher values of zonulin (*n* = 13, 10.36 ± 3.32) compared to SLE patients receiving HCQ combined with Mycophenolate mofetil (MMF) (*n* = 3, 7.31 ± 5.65), compared to the group of SLE patients receiving HCQ combined with AZA (*n* = 6, 5.193 ± 1.473), or compared to SLE patients with AZA administration alone (*n* = 2, 2.88 ± 2.46) or Belimumab administration alone (*n* = 2, 2.03 ± 0.29).

Furthermore, the Mann–Whitney test showed no statistically significant differences between patients receiving GC therapy combined with HCQ compared to those receiving HCQ alone (*n* = 13, 10.36 ± 3.32 vs. *n* = 5, 4.326 ± 1.561, *p* = 0.502). Similarly, there were no significant differences between patients treated with GCs combined with HCQ and AZA compared to those receiving only HCQ combined with AZA (*n* = 6, 5.193 ± 1.473 vs. *n* = 4, 13.66 ± 6.323, *p* = 0.257), or between those treated with GCs combined with Belimumab versus those receiving Belimumab alone (*n* = 2, 2.030 ± 0.29 vs. *n* = 2, 1.61 ± 1.12, *p* > 0.999).

## 4. Discussion

The first important finding of our study was that the Romanian patients diagnosed with SLE exhibited significantly elevated plasma levels of zonulin compared to the control group, indicating an increased intestinal permeability in SLE. All the subjects included in our study belonged to the Caucasian race.

The present results are similar to those found in an earlier study performed in Portugal [19], but different from those of one conducted in the USA, according to which no significant difference in zonulin levels was observed between White patients with SLE and White controls [20]. Nevertheless, in the same paper, higher zonulin levels were found in Black patients compared to Black controls. Also, SLE patients had higher levels of lipopolysaccharide (LPS) when compared to healthy controls, irrespective of race, a fact that reveals the presence of plasma microbial translocation in the case of SLE [20]. Scientists suggested that the racial differences in plasma microbiome (e.g., lower microbial α-diversity in Black subjects) associated with the disrupted epithelial intestinal barrier in Black patients may be correlated with the well-known higher severity of SLE experienced by Black patients, when compared with White patients [20]. Similarly, we speculate that potential differences in gut microbiome between Romanian subjects and USA white subjects may be at least partially responsible for this apparent non-concordance between the results of our study and the USA study. Moreover, previous evidence suggests that gut microbiome composition differs across countries. These variations might be derived from various factors, such as dietary choices, lifestyle, ethnicity, environmental biodiversity as a result of urbanization, and geographical location [21].

In another study, zonulin levels were also found to be significantly higher in the fecal samples of SLE patients when compared with those of healthy individuals [22].

The mechanisms leading to the loss of intestinal permeability in patients with SLE are complex. The reduction in populations of short-chain fatty acid-producing bacteria, along with the abnormal expansion of *Ruminococcus gnavus*, a mucin-degrading bacterium, results in increased intestinal permeability by disrupting the mucus layer of the intestinal barrier. Additionally, immune system hyperactivity leads to chronic inflammation and the recruitment of inflammatory cells in the intestine. Proinflammatory cytokines involved in the pathogenesis of SLE (TNF-α, IL-1β, INFγ) compromise the integrity of the intestinal barrier. Moreover, certain medications used in the treatment of SLE, such as non-steroidal anti-inflammatory drugs, can impair the function of the intestinal barrier [11].

Other biomarkers of enhanced intestinal permeability were also studied in SLE. One study found higher levels of sCD14 in SLE, as well as intestinal fatty acid-binding protein (I-FABP), but only in adult patients [19], whereas another reported that only sCD14 was increased in SLE patients, particularly in those with lupus nephritis [23]. All these results reflect increased intestinal permeability in SLE. Furthermore, in the latter study, plasma sCD14 levels were positively correlated with the disease activity (assessed by SLEDAI score) and proteinuria level [23].

A compromised intestinal barrier permits foreign antigens, like LPS and 1,3-β-D-glucan, to enter the systemic circulation. 1,3-β-D-glucan, a component of the fungal cell wall, has been identified in the serum of individuals with lupus nephritis [24]. These microbial antigens are known to induce the secretion of proinflammatory cytokines, including type I INF, through the activation of dectin-1 by 1,3-β-D-glucan and the activation of TLR-4 by LPS. Both of these antigens have been identified in the sera of patients with SLE [10,13,25]. Furthermore, systemic administration of lipoteichoic acid, a key cell wall component of Gram-positive bacteria, has been shown to induce SLE-type anticardiolipin antibodies in rabbits [26].

According to our results, plasma zonulin levels showed no statistical difference with the patient age, geographical background, or smoking status. Consequently, we suggest that the integrity of the intestinal barrier may not be influenced by these demographic factors (age or geographical background) or smoking, and the disruption of this barrier may be a common trait of patients with SLE.

Interestingly, oral administration of Larazotide acetate, a zonulin antagonist, restored intestinal gut permeability in mice colonized with a *Ruminococcus gnavus* strain obtained from SLE patients, suggesting that Larazotide acetate could serve as a potential therapeutic intervention to ameliorate gut barrier function in SLE [11,27].

A similar relationship between zonulin levels and other autoimmune diseases was reported. For instance, patients with spondylarthritis exhibited elevated serum zonulin levels compared to control groups, suggesting that leaky gut syndrome may induce a systemic immune response, potentially contributing to the pathogenesis of spondylarthritis [28]. Moreover, elevated markers of intestinal permeability were found to be present before the clinical onset of rheumatoid arthritis, leading to the idea that serum zonulin may be considered as a potential risk factor for developing the disease in predisposed individuals [29]. Furthermore, larazotide improved symptoms in mice with collagen-induced arthritis [29].

In terms of disease activity, measured by SLEDAI score, there was no significant correlation with plasma zonulin levels. This finding was similar to the results obtained from a previous study performed in Portugal [19], but in contradiction with the results of another study conducted in the USA, according to which zonulin levels were positively correlated with disease activity in female lupus patients [30].

In order to perform a detailed analysis of the variability within the patient group, a larger number of subjects were included in the SLE group, while the control group served mainly as a reference and required fewer cases. The difference in the number of subjects between the two groups (LES versus control) also resulted from the multiple variables matching procedure. Besides these methodological reasons, the distribution of subjects between the two groups was also influenced by logistical factors, as the selection of healthy volunteers was constrained by their availability and the limited study period, while the patients included in the study were already hospitalized and thus more easily accessible.

No statistically significant relationship was observed in our study between organ involvement and plasma zonulin levels among the analyzed subgroups, with one exception, which seems to be another important finding of our study: patients without articular involvement demonstrated significantly elevated plasma levels of zonulin (*n* = 7, 17.1 ± 4.529 vs. *n* = 34, 5.604 ± 1.134, *p* < 0.01). This result seems to be paradoxical, considering the concept of the “gut-joint axis” described in the literature [31]. According to this concept, the pathogenesis of joint involvement is dependent on the inflammatory state associated with the increased intestinal permeability [32].

The concept of the “gut-joint axis” has gained substantial attention in recent medical research trying to elucidate the relationship between intestinal permeability, intestinal microbiota, and clinical manifestations in rheumatic diseases. Disruption of intestinal barrier integrity and the subsequent increase in intestinal permeability may explain how immune cells from the gut migrate to the joints, a process also known as gut–joint iteropathy (iter-journey). The iteropathy concept was introduced by Sheldon in 1988 [33]. He suggested that the synovial membrane (similarly with the gut-associated lymphoid tissue, GALT, bronchi-associated lymphoid tissue, and genitourinary tract lymphoid tissue) should also be considered as a part of the secretory immune system [33]. Thus, gut–joint iteropathy may represent a pathological migratory behavior of lymphocytes, which operate within the boundaries of this complex, widespread system. Nevertheless, despite some progress in understanding the molecular mechanisms of this abnormality, there are still many aspects to be revealed.

An argument supporting the existence of lymphocyte gut–joint itineraries is represented by the discovery of identical T cell clones in both the joints and intestines of individuals with spondylarthritis [34]. Furthermore, T cells expressing the intestinal receptor integrin αEβ7 were observed in the synovium of patients with rheumatoid arthritis [35]. Activated B cells originating in the intestine exhibit a marked adherence to endothelial venules in both the intestinal mucosa and synovial tissue, though this adhesion does not extend to peripheral lymph nodes [36].

The intriguingly higher values of zonulin in patients without articular involvement encourage us to find potential explanations and lead us to a new hypothesis. We suggest that the gut–joint iteropathy may be bidirectional: lymphocytes migrate from the gut to the joints, but also, they may return to the gut. Thus, the inflammatory “signature” appears either in the gut or in the joints, depending on the site where lymphocytes are predominantly dislocated within the immune system. Quite interestingly, lymphocytes move in and out of the lymphoid tissues at the level of high endothelial venules, which are present in GALT, but also in the synovial membrane [33].

The higher values of zonulin in SLE subjects without articular involvement may also suggest a paradoxical protective role of zonulin against articular tissue injuries that should be further explored. The biological effects of zonulin in extra-intestinal locations are unknown, with few exceptions (e.g., intrapulmonary activation of complement) [37].

Regarding the treatment, the lack of significant difference in zonulin concentrations between patients taking or not taking corticosteroids was in concordance with the results reported in a previous study [30].

Considering the absence of statistically significant variations in plasma zonulin level among both glucocorticoid-treated and non-glucocorticoid-treated groups, regardless of the concomitant medications, we can conclude that the medication used in patients with SLE does not appear to influence the integrity of the intestinal permeability, as indicated by plasma zonulin levels.

There are a number of limitations to this study, like the relatively reduced number of participants and the heterogeneity of the treatment in the SLE group. Also, the patient sample included subjects with a long duration of disease (more than 8–9 years). The results may not be valid when another temporal framing would be used (e.g., patients with a shorter duration of SLE). The present results should be interpreted with caution, since this is a cross-sectional study, and therefore it is difficult to make causal inferences between the leaky gut and the development of SLE. Moreover, zonulin is also expressed in extra-intestinal organs (e.g., heart, brain) [38]; therefore, its serum level significance should be carefully interpreted.

## 5. Conclusions

In conclusion, our study revealed that the level of plasma zonulin, a protein that plays a crucial role in regulating the integrity of the intestinal barrier, was higher in patients with SLE compared to healthy individuals (*p* < 0.01). This fact suggests that an increased intestinal permeability in SLE patients may contribute to the pathogenesis of the disease, although this did not correlate with disease activity and was not demonstrated to be relevant in terms of organ involvement, with one exception. Intestinal permeability was paradoxically found to be higher in SLE patients without articular involvement than in those with articular manifestations (*p* < 0.01), suggesting a potential mutually exclusive role between intestinal barrier damage and articular damage.

The observed pattern of plasma zonulin levels—characterized by lower concentrations in the control group compared to the SLE one, higher levels in SLE patients without clinical articular involvement, and moderately elevated levels in those with joint manifestations—suggests that a progressive decline in plasma zonulin concentration upon serial measurements may represent a potential predictive biomarker for the onset of clinical articular involvement in SLE. This finding highlights the potential clinical significance of regular monitoring of plasma zonulin levels as part of disease surveillance.

Nevertheless, further studies performed on a higher number of patients should confirm the present findings.

Considering our results, we assume that other interesting directions to be explored in the future would be (1) to evaluate whether interventions based on the modulation of intestinal permeability (e.g., zonulin antagonist) may influence the course of the SLE disease and (2) to conduct serial measurements of multiple inflammatory mediators (e.g., TNF-α, IFN-γ) in serum samples and to correlate these parameters with circulating zonulin levels and the degree of articular involvement.

## Figures and Tables

**Table 1:** Demographic characteristics (age, gender, place of origin), smoking status, and presence of anxiety/depression in patients with SLE and the control group.

Variable	SLE Patients	Control Group	*p* Values
Number	41	29	NA
Sex (F/M)	40/1	26/3	0.37
Age (years)	44.17 ± 1.966	49.72 ± 2.39	0.076
Place of origin (R/U)	16/25	5/24	0.065
Smoker/former smoker/non-smoker	7/6/28	5/2/22	0.683
Anxiety/depression	9	2	0.1

Age was represented as mean ± standard error of the mean; R—rural, U—urban.

**Table 2:** Clinical characteristics of patients with SLE.

Variable	SLE Patients
Disease duration since symptoms onset (years)	10.71 ± 1.563
Disease duration since diagnosis (years)	9.512 ± 1.491
Neurological involvement	8
Renal involvement	11
Cutaneous involvement	26
Articular involvement	34
Hematological involvement	30
Personal history of serositis	10
Secondary Sjogren’s syndrome	22
SLEDAI	4.863 ± 0.605

Disease duration since symptoms onset, disease duration since diagnosis and SLEDAI are represented as mean ± standard error of the mean; SLE Disease Activity Index—SLEDAI.

**Table 3:** Laboratory parameters of patients with SLE.

Variable	SLE Patients (Mean ± SEM)
Plasma zonulin level (ng/mL)	7.566 ± 1.368
C3 plasma level (mg/dL)	90.53 ± 3.372
C4 plasma level (mg/dL)	12.36 ± 1.296
Anti-dsDNA ab (UI/mL)	72.26 ± 11
ESR (mm/H)	22.61 ± 3.023
CRP (mg/L)	10.23 ± 3.243
Creatinine (mg/dL) *	1.093 ± 0.172
24 h urine protein (mg/24 h) Δ	282.9 ± 90.73
WBC (×10^3^/mm^3^)	6.607 ± 0.497
LYM (×10^3^/mm^3^)	1.383 ± 0.143
PLT (×10^3^/mm^3^)	257.6 ± 13.21
Hb (g/dL)	12.36 ± 0.21
Serum uric acid level (mg/dL) †	4.254 ± 0.151

Results are presented as mean ± standard error of the mean. C3—complement component 3, C4— complement component 4, Anti-dsDNA ab—anti-double stranded DNA antibodies, ESR—erythrocyte sedimentation rate, CRP—C reactive protein, WBC—white bloods cells, LYM—lymphocytes, PLT—platelets, Hb—hemoglobin; * Absence of data for 1 patient; † Absence of data for 6 patients; Δ Absence of data for 8 patients.

## Data Availability

The original contributions presented in this study are included in the article. Further inquiries can be directed to the corresponding author.

## Abbreviations

The following abbreviations are used in this manuscript:

SLE	Systemic Lupus Erythematosus
SLEDAI	Systemic Lupus Erythematosus Disease Activity Index
ESR	Erythrocyte sedimentation rate
CRP	C-reactive protein
C3	Complement component C3
C4	Complement component C4
Anti-dsDNA ab	Anti-double-stranded DNA antibodies
WBC	White blood cells
LYM	Lymphocytes
PLT	Platelets
Hb	Hemoglobin

## References

[1] Zucchi D., Silvagni E., Elefante E., Signorini V., Cardelli C., Trentin F., Schilirò D., Cascarano G., Valevich A., Bortoluzzi A. (2023). Systemic lupus erythematosus: One year in review 2023. Clin. Exp. Rheumatol..

[2] Langefeld C.D., Ainsworth H.C., Graham D.S.C., Kelly J.A., Comeau M.E., Marion M.C., Howard T.D., Ramos P.S., Croker J.A., Morris D.L. (2017). Transancestral mapping and genetic load in systemic lupus erythematosus. Nat. Commun..

[3] Cornet A., Andersen J., Myllys K., Edwards A., Arnaud L. (2021). Living with systemic lupus erythematosus in 2020: A European patient survey. Lupus Sci. Med..

[4] Rider V., Abdou N.I., Kimler B.F., Lu N., Brown S., Fridley B.L. (2018). Gender Bias in Human Systemic Lupus Erythematosus: A Problem of Steroid Receptor Action?. Front. Immunol..

[5] Peterson L.W., Artis D. (2014). Intestinal epithelial cells: Regulators of barrier function and immune homeostasis. Nat. Rev. Immunol..

[6] Diwan M.A., Lahimer M., Bach V., Gosselet F., Khorsi-Cauet H., Candela P. (2023). Impact of Pesticide Residues on the Gut-Microbiota–Blood–Brain Barrier Axis: A Narrative Review. Int. J. Mol. Sci..

[7] Bist P., Choudhary S. (2022). Impact of Heavy Metal Toxicity on the Gut Microbiota and Its Relationship with Metabolites and Future Probiotics Strategy: A Review. Biol. Trace Elem. Res..

[8] Shen W., Zhao M., Xu W., Shi X., Ren F., Tu P., Gao N., Shan J., Gao B. (2024). Sex-Specific Effects of Polystyrene Microplastic and Lead(II) Co-Exposure on the Gut Microbiome and Fecal Metabolome in C57BL/6 Mice. Metabolites.

[9] Aleman R.S., Moncada M., Aryana K.J. (2023). Leaky Gut and the Ingredients That Help Treat It: A Review. Molecules.

[10] Thim-Uam A., Surawut S., Issara-Amphorn J., Jaroonwitchawan T., Hiengrach P., Chatthanathon P., Wilantho A., Somboonna N., Palaga T., Pisitkun P. (2020). Leaky-gut enhanced lupus progression in the Fc gamma receptor-IIb deficient and pristane-induced mouse models of lupus. Sci. Rep..

[11] Ma L., Morel L. (2022). Loss of Gut Barrier Integrity in Lupus. Front. Immunol..

[12] Ogunrinde E., Zhou Z., Luo Z., Alekseyenko A., Li Q., Macedo D., Kamen D.L., Oates J.C., Gilkeson G.S., Jiang W. (2019). A Link Between Plasma Microbial Translocation, Microbiome, and Autoantibody Development in First-Degree Relatives of Systemic Lupus Erythematosus Patients. Arthritis Rheumatol..

[13] Shi L., Zhang Z., Yu A.M., Wang W., Wei Z., Akhter E., Maurer K., Reis P.C., Song L., Petri M. (2014). The SLE Transcriptome Exhibits Evidence of Chronic Endotoxin Exposure and Has Widespread Dysregulation of Non-Coding and Coding RNAs. PLoS ONE.

[14] Fasano A. (2012). Zonulin, regulation of tight junctions, and autoimmune diseases. Ann. N. Y. Acad. Sci..

[15] Fasano A., Not T., Wang W., Uzzau S., Berti I., Tommasini A., Goldblum S.E. (2000). Zonulin, a newly discovered modulator of intestinal permeability, and its expression in coeliac disease. Lancet.

[16] Tripathi A., Lammers K.M., Goldblum S., Shea-Donohue T., Netzel-Arnett S., Buzza M.S., Antalis T.M., Vogel S.N., Zhao A., Yang S. (2009). Identification of human zonulin, a physiological modulator of tight junctions, as prehaptoglobin-2. Proc. Natl. Acad. Sci. USA.

[17] Fasano A. (2011). Zonulin and Its Regulation of Intestinal Barrier Function: The Biological Door to Inflammation, Autoimmunity, and Cancer. Physiol. Rev..

[18] Aringer M. (2019). EULAR/ACR classification criteria for SLE. Semin. Arthritis Rheum..

[19] Almada-Correia I., Castro M., Motta C., Guerreiro C.S., Moura R.A., Khmelinskii N., Oliveira-Ramos F., Barreto G., Eklund K., Mendes C.I. (2023). UNRAVELING THE MYSTERIES OF THE CONNECTION BETWEEN GUT AND THE CHRONIC ACTIVATION OF THE IMMUNE SYSTEM IN SYSTEMIC LUPUS ERYTHEMATOSUS. Ann Rheum Dis..

[20] Wen X., Ogunrinde E., Wan Z., Cunningham M., Gilkeson G., Jiang W. (2024). Racial Differences in Plasma Microbial Translocation and Plasma Microbiome, Implications in Systemic Lupus Erythematosus Disease Pathogenesis. ACR Open Rheumatol..

[21] Tasnim N., Abulizi N., Pither J., Hart M.M., Gibson D.L. (2017). Linking the Gut Microbial Ecosystem with the Environment: Does Gut Health Depend on Where We Live?. Front. Microbiol..

[22] Gudi R., Kamen D., Vasu C. (2022). Fecal immunoglobulin A (IgA) and its subclasses in systemic lupus erythematosus patients are nuclear antigen reactive and this feature correlates with gut permeability marker levels. Clin. Immunol..

[23] Panda A.K., Tripathy R., Das B.K. (2020). CD14 (C-159T) polymorphism is associated with increased susceptibility to SLE, and plasma levels of soluble CD14 is a novel biomarker of disease activity: A hospital-based case-control study. Lupus.

[24] Issara-Amphorn J., Surawut S., Worasilchai N., Thim-Uam A., Finkelman M., Chindamporn A., Palaga T., Hirankarn N., Pisitkun P., Leelahavanichkul A. (2018). The Synergy of Endotoxin and (1→3)-β-D-Glucan, from Gut Translocation, Worsens Sepsis Severity in a Lupus Model of Fc Gamma Receptor IIb-Deficient Mice. J. Innate Immun..

[25] Takeuchi O., Akira S. (2010). Pattern Recognition Receptors and Inflammation. Cell.

[26] Gotoh M., Matsuda J. (1996). Induction of anticardiolipin antibody and/or lupus anticoagulant in rabbits by immunization with lipoteichoic acid, lipopolysaccharide and lipid A. Lupus.

[27] Deng J., Azzouz D.F., Ferstler N., Silverman G.J. (2021). Sex-dependent Lupus *Ruminococcus blautia gnavus* strain induction of zonulin-mediated intestinal permeability and autoimmunity. bioRxiv.

[28] Ciccia F., Guggino G., Rizzo A., Alessandro R., Luchetti M.M., Milling S., Saieva L., Cypers H., Stampone T., Di Benedetto P. (2017). Dysbiosis and zonulin upregulation alter gut epithelial and vascular barriers in patients with ankylosing spondylitis. Ann. Rheum. Dis..

[29] Tajik N., Frech M., Schulz O., Schälter F., Lucas S., Azizov V., Dürholz K., Steffen F., Omata Y., Rings A. (2020). Targeting zonulin and intestinal epithelial barrier function to prevent onset of arthritis. Nat. Commun..

[30] Bowes M.M., Casares-Marfil D., Sawalha A.H. (2024). Intestinal permeability correlates with disease activity and DNA methylation changes in lupus patients. Clin. Immunol..

[31] Xu X., Wang M., Wang Z., Chen Q., Chen X., Xu Y., Dai M., Wu B., Li Y. (2022). The bridge of the gut–joint axis: Gut microbial metabolites in rheumatoid arthritis. Front. Immunol..

[32] Blenkinsopp H.C., Seidler K., Barrow M. (2023). Microbial Imbalance and Intestinal Permeability in the Pathogenesis of Rheumatoid Arthritis: A Mechanism Review with a Focus on Bacterial Translocation, Citrullination, and Probiotic Intervention. J. Am. Nutr. Assoc..

[33] Sheldon P. (1988). Rheumatoid arthritis and gut related lymphocytes: The iteropathy concept. Ann. Rheum. Dis..

[34] May E., Märker–Hermann E., Wittig B.M., Zeitz M., Büschenfelde K.M.Z., Duchmann R. (2000). Identical T-cell expansions in the colon mucosa and the synovium of a patient with enterogenic spondyloarthropathy. Gastroenterology.

[35] Zaiss M.M., Wu H.-J.J., Mauro D., Schett G., Ciccia F. (2021). The gut–joint axis in rheumatoid arthritis. Nat. Rev. Rheumatol..

[36] Trollmo C., Verdrengh M., Tarkowski A. (1997). Fasting enhances mucosal antigen specific B cell responses in rheumatoid arthritis. Ann. Rheum. Dis..

[37] Rittirsch D., Flierl M.A., Nadeau B.A., Day D.E., Huber-Lang M.S., Grailer J.J., Zetoune F.S., Andjelkovic A.V., Fasano A., Ward P.A. (2013). Zonulin as prehaptoglobin2 regulates lung permeability and activates the complement system. Am. J. Physiol. Cell. Mol. Physiol..

[38] Wang W., Uzzau S., Goldblum S.E., Fasano A. (2000). Human zonulin, a potential modulator of intestinal tight junctions. J. Cell Sci..

